# Is Nature Relatedness Associated with Better Mental and Physical Health?

**DOI:** 10.3390/ijerph15071371

**Published:** 2018-06-29

**Authors:** Julie H. Dean, Danielle F. Shanahan, Robert Bush, Kevin J. Gaston, Brenda B. Lin, Elizabeth Barber, Lara Franco, Richard A. Fuller

**Affiliations:** 1 Faculty of Medicine, University of Queensland, Brisbane, QLD 4006, Australia; cphcrobert@gmail.com (R.B.); e.barber@uq.edu.au (E.B.); 2 School of Biological Sciences, University of Queensland, Brisbane, QLD 4072, Australia; danielle.shanahan@visitzealandia.com (D.F.S.); l.franco@uq.edu.au (L.F.); r.fuller@uq.edu.au (R.A.F.); 3 Zealandia, 31 Waiapu Road, Karori, WLG 6012, New Zealand; 4 Environment & Sustainability Institute, University of Exeter, Penryn, Cornwall TR10 9EZ, UK; k.j.gaston@exeter.ac.uk; 5 CSIRO Land & Water Flagship, PMB 1, 107-121 Station Street, Aspendale, VIC 3195, Australia; Brenda.Lin@csiro.au

**Keywords:** nature relatedness, depression, anxiety, stress, health

## Abstract

Nature relatedness is a psychological characteristic with the potential to drive interaction with nature and influence well-being. We surveyed 1538 people in Brisbane, Australia to investigate how nature relatedness varies among socio-demographic groups. We determined whether people with higher nature relatedness reported fewer symptoms of depression, anxiety, stress and better overall health, controlling for potentially confounding socio-demographic and health-related variables. Overall nature relatedness was higher in older people, females, those without children living at home, not working, and people speaking English at home. Aspects of nature relatedness reflecting enjoyment of nature were consistently associated with reduced ill health, consistent with widespread evidence of the health and well-being benefits of experiencing nature. In contrast, aspects of nature relatedness reflecting self-identification with nature, and a conservation worldview, were associated with increased depression, anxiety or stress, after accounting for potential confounding factors. Detailed investigation of causal pathways among nature relatedness, socio-demographic factors and health is warranted, with particular focus on the relationship between stress and nature orientation.

## 1. Introduction

Human society has become increasingly urbanized over the past century with more than half of the world’s population now living in towns and cities [1]. As a result, many people now have fewer opportunities to interact with nature, defined as landscapes with living organisms, including plants and non-human animals [2]. There is mounting concern that this ‘extinction of experience’ of nature [3] has led to nature becoming less relevant in people’s lives as well as having negative impacts on human health and well-being [3,4,5,6,7,8]. The concept of nature relatedness—individual differences in the way people view the natural world and their connection with nature—has become the focus of increasing attention as an individual-level characteristic that may drive interaction with nature and influence well-being [2,9,10,11]. Yet, there is limited empirical data on the connection between nature relatedness and mental and physical ill health. This paper contributes to knowledge about the psychological construct of nature relatedness through: (1) examining differences in nature relatedness in people from different socio-demographic backgrounds; (2) investigating the relationships between nature relatedness and mental and physical ill health when accounting for socio-demographic characteristics and other key health-related variables; and (3) systematically considering these relationships across multiple dimensions of nature relatedness reflecting affective, cognitive and experiential aspects.

### 1.1. Benefits from Contact with Nature

There is well documented evidence that when humans interact with nature they tend to experience benefits, including physiological and psychological stress reduction [12,13,14,15], increases in well-being [16,17,18]; vitality [19], restored capacity to direct attention to relevant tasks in daily life [20], ability to reflect on a life problem [18] and decreased rumination and neural activity in an area of the brain associated with behavioral withdrawal [21]. Places with natural features can provide a setting for behaviors that enhance health and well-being, such as physical activity [22,23] and social contact [24,25,26]. Indeed, a number of these pathways may be simultaneously activated when a person has contact with a natural setting [8,27].

There is immense potential to develop effective public health interventions that harness people’s interactions with, and orientations to, urban nature as a pathway to promote health [28]. Globally, the leading cause of years of life lost is chronic disease associated with urbanization and sedentary lifestyles [29]. The leading cause of non-fatal burden of disease is mental ill health [30] and there is high co-morbidity for chronic disease and mental ill health [31]. The health benefits of interacting with urban nature—including promoting physical activity, psychological restoration, reduced stress and enhanced social connection—have the potential to reduce the burden of the major non-communicable diseases, such as cardiovascular diseases and some forms of diabetes, that are prevalent in high and middle income countries [32]. Further, careful design and provision of green space holds promise as one approach to reducing ill health at the population level [33].

### 1.2. The Role of Nature Relatedness in Enhancing Health

Across centuries and societies the connections between nature and human well-being have formed part of the cultural fabric in stories, art, poetry and other traditional practices [34]. Recent theorizing has sought to explain why people experience positive emotions when in contact with natural features. The two main explanatory frameworks are stress reduction theory and attention restoration theory. Stress reduction theory, located within psycho-evolutionary theory, posits that when individuals are in contact with natural elements that support survival (e.g., water, food etc.) they experience a psychophysiological response involving a decrease in physiological arousal, reduced negative affect and increased positive affect [35]. Attention restoration theory proposes that contact with nature helps replenish ‘directed attention’—a form of focused concentration that can become depleted in urban environments—through unconscious cognitive processes [36]. Several related explanations have emerged from these frameworks, for example positive responses to nature contact being related to ‘processing fluency’ whereby the visual structure of nature settings requires fewer cognitive resources than for urban settings [37] and the ‘topophilia hypothesis’ in which the co-evolution of innate behavior and cultural learning fosters connection with nature through attachment to place [38]. Psychological theories of identity have posited the connection between well-being and nature through the concept of ‘ecological identity’ or sense of ‘ecological self’, experienced when one’s sense of self is expanded to include not just other humans but other living species and ecosystems [39,40]. Ecological philosopher Arne Naess proposed that an expanded sense of oneself as in a reciprocal relationship with other living species and ecological systems, rather than a more independent constrained sense of self, can provide an enhanced sense of self-realization and fulfillment of personal potential [40].

Over the previous decade there has been growing empirical research on individual differences in such connections to nature, with the development of a number of scales sharing a common construct [41]. Measures of nature relatedness assess various dimensions of this construct including emotional affiliation (e.g., feelings of oneness with nature), cognitive processes (e.g., views about how nature overlaps with one’s sense of self), relationship commitment (e.g., feelings of attachment to nature), as well as personal experience and behavior (e.g., time spent in nature) [41]. Connection to nature is typically considered as trait-like, or relatively stable over time for an individual, although it can fluctuate over time in response to changed contexts [42,43,44]. In this paper, we use the term ‘nature relatedness’ to refer to the wider psychological construct encompassing connectedness and relationships with nature.

### 1.3. Is Strong Nature Relatedness Protective Against Ill Health?

Research on nature relatedness has typically focused on relationships with indicators of positive psychological health, rather than psychological or physical ill health. In general, people scoring higher on a measure of connection to nature are more likely to report higher levels of subjective well-being (concerning optimizing pleasure and minimizing pain [41,42,43,44,45]) and psychological well-being (relating to self-actualization and fulfilling one’s potential [46]), although occasionally no association is found [47]. Zelenski and Nisbet (2014) found that nature relatedness was more likely to correlate with indicators of positive well-being than indicators of negative mental health (specifically negative affect and symptoms of depression), proposing that nature relatedness may have a more important role in promoting well-being than buffering negative psychological health [48]. There is inconsistent evidence of relationships between nature relatedness and mental ill health. Several studies have reported correlations between some measures of nature relatedness and reduced anxiety and depression [49,50,51], and a measure of nature relatedness concerning familiarity with and experiences in nature predicted lower somatic anxiety [50]. In contrast, non-significant relationships between nature relatedness and some mental ill-health measures have been reported when accounting for other participant characteristics [49,50]. Here we focus on indicators of psychological and physical ill health to explore the potential role of nature relatedness as a protective factor against ill health.

### 1.4. Differences in Nature Relatedness Among Social Groups

Understanding differences in nature relatedness across socio-demographic groups can help shed light on potential influencing factors underpinning the development of this orientation. While the personal and social factors influencing environmental concern and behavior have been a focus for investigation [52], to our knowledge socio-demographic factors have not been systematically investigated for the construct of nature relatedness. Additionally, accounting for the effects of socio-demographic variables when considering the relationship between nature relatedness and ill health is critical given the extensive literature on the influence of social characteristics on health. Social characteristics influence health and well-being through complex causal chains of mediating factors, where social, economic and political processes create social hierarchies, which in turn shape the conditions of daily life that determine health status [53]. Key social characteristics associated with mental and physical health include socio-economic disadvantage, gender, cultural background, age and life stage [30,54]. Several studies have reported that health benefits of green space vary according to these kinds of socio-demographic factors, with greater benefits variously reported for young people, older people, those who are socioeconomically disadvantaged, and men [55,56]. Associations between one or several socio-demographic characteristics and nature relatedness have been reported, for example women and older people have scored higher than men and younger age groups [47,57,58]. However, in studies accounting for the influence of several characteristics such as gender and age, these variables were not significant moderators of relationships between nature relatedness and various indicators of well-being [18,45,58]. If higher nature relatedness is associated with reduced psychological and physical ill health after accounting for the effects of a range of socio-demographic variables, this suggests a potential role for enhancing nature relatedness in public health initiatives.

Here we address a critical gap in knowledge about the role of nature relatedness in fostering health. The first step in this paper is to consider the associations between nature relatedness and a broad range of socio-demographic factors. We then consider the relationships between nature relatedness and four variables related to ill health—depression, anxiety, stress and self-reported health—while accounting for a broad range of potentially confounding socio-demographic indicators and other health-related variables in a large community sample. The findings will shed light on the potential for nature relatedness to be considered as a psychological orientation relevant to the development, implementation and evaluation of public health initiatives.

## 2. Materials and Methods

### 2.1. Study Location

This study was undertaken in Brisbane, Queensland, a city located in subtropical eastern Australia. The local government authority area covers 1380 km^2^, with a human population of approximately 1,090,000 in 2011. A substantial per capita area of land is designated as public green space within the city (over 200 m^2^ per person, with these areas including, for example, manicured parks, natural bushland, and children’s play areas). This green space is relatively evenly distributed across the built up part of the city [11].

### 2.2. Participants and Procedure

An online survey was delivered to 1538 respondents across Brisbane in November 2012 (late spring) via Q&A Market Research Ltd (Brisbane, Australia). Survey respondents were part of a market research panel through which they receive “points” for completing surveys they have been invited to fill-in. This approach allowed stratification of the survey population, which was completed by people between 18 and 70 years of age stratified such that the sample spanned variation among the population, being: (a) equally spread across male and females; (b) equally spread above and below 45 years of age; (c) equally sampling income quartiles representative of the total Brisbane population (2011 Australian Census data); and (d) equally sampling respondents residing across four spatial zones reflecting quartiles of different levels of tree cover across the city. Participants were invited to complete a 15–20 min survey considering how city spaces influence lifestyles and well-being (for full scale see [59]).

### 2.3. Measures

#### 2.3.1. Nature Relatedness

The Nature Relatedness (NR) Scale measures a person’s affective, cognitive and experiential relationships with the natural world on a 21 item scale (for complete scale see [43]). We used this scale due to its sound psychometric properties and multidimensional nature, comprising three subscales assessing different aspects of a person’s relationship with nature: NR Self—assessing personal identification and connection with nature (8 items; e.g., “My relationship with nature is an important part of who I am”; Cronbach’s alpa = 0.85); NR Perspective—assessing a person’s pro-environmental conservation attitudes, world view and sense of agency relating to nature including the impacts of humans on all living things (7 items; e.g., “Conservation is unnecessary because nature is strong enough to recover from any human impact” (reversed; Cronbach’s alpha = 0.51); humans have the right to use natural resources any way we want (reversed; Cronbach’s alpha = 0.52); and NR Experience—assessing a person’s attraction to and physical familiarity with nature (6 items; e.g., “I enjoy being outdoors, even in unpleasant weather”; Cronbach’s alpha = 0.76). Tam (2013) found that the Nature Relatedness Scale tended to be more highly correlated with criterion variables such as personality traits, subjective well-being and environmental behavior than other unidimensional measures, as well as having an additional ability to predict some criterion variables [41], although Capaldi et al. (2014) found the converse, where the single item inclusion of nature in self measure had the strongest relationship with indicators of happiness in comparison to other measures [45]. Each item on the NR scale is presented on a five point Likert scale from 1 (disagree strongly) to 5 (agree strongly), with higher scores indicating stronger connection to nature (8 items were reverse scored). The NR Score is the mean of the responses to the 21 statements, with subscale scores being the mean of the responses to the items in each subscale. Nature relatedness can therefore vary between 1 and 5.

#### 2.3.2. Depression, Anxiety and Stress

Respondents completed the Depression Anxiety Stress Scales-21 (DASS-21 [60]). Each subscale consisted of seven items, with lower scores indicative of lower levels of depression, anxiety or stress. This scale was developed and validated with a range of population samples across different nations and in both clinical and nonclinical settings [61,62,63]. Respondents rated each symptom regarding its severity during the previous week on a four point response scale from 0 (did not apply to me at all) to 3 (applied to me very much or most of the time). The total score for each of the subscales was used for analysis purposes in this study (Cronbach’s alpha for depression subscale = 0.93, anxiety subscale = 0.90, and stress subscale = 0.88).

#### 2.3.3. Self-Reported Health

Respondents were asked to rate their own health on a five point rating with five response options (from very poor to very good). There is extensive evidence that this single item measure designed to reduce burden for respondents is significantly associated with a wide range of health-related outcomes including mortality, changes in health status, health service usage, and experience of and recovery from specific health problems, thus providing a robust measure of current health status [64].

#### 2.3.4. Socio-Demographic Indicators

Respondents provided information on key socio-demographic characteristics (outlined in Table S1) including age, gender, educational attainment, personal annual income, how many hours a week they usually worked and primary language spoken at home. Respondents also indicated whether any children under 16 lived at home.

#### 2.3.5. Other Health-Related Variables

Respondents indicated the number of days they exercised for 30 min or more during the previous week, with the final score being between 0 and 7. Body Mass Index (BMI) was determined from respondent self-reports of height and weight, calculating BMI as weight in kilograms divided by the square of height in meters [65]. Perceptions of social cohesion were assessed using 3 scales (17 items) measuring trust, reciprocal exchange within communities, and general community cohesion. The items included five from Sampson, Raudenbush, and Earls’ (1997) social cohesion and trust scale (e.g., “People in this community are willing to help their neighbors” with response options ‘Don’t know’, ‘Disagree strongly’, ‘Disagree’, ‘Agree’, ‘Agree strongly’; Cronbach’s alpha = 0.82) [66], six items adapted from the reciprocated exchange scale developed by Sampson, Morenoff, and Earls (1999; e.g., “About how often do you and people in your community do favors for each other?” with response options ‘Don’t know’, ‘Never’, ‘Rarely’, ‘Sometimes’, ‘Often’; Cronbach’s alpha = 0.89) [67] and six items from a general measure of social capital using components from Bullen and Onyx (1998; e.g., “Do you think multiculturalism makes life in your area better?” with response options ‘Don’t know’, ‘Not at all’, ‘Not often’, ‘Sometimes’, or ‘Yes, definitely’; Cronbach’s alpha = 0.68) [68]. ‘Don’t know’ responses were excluded from the analysis. An average score was generated for each of the three scales, with higher scores indicating greater social cohesion (Cronbach’s alpha across all 17 items was 0.81). The average of scores from the three scales provided an overall estimate of social cohesion resulting in a continuous score from highest (4) to the lowest (0) perceived social cohesion.

### 2.4. Ethical Clearance

This research was conducted in accordance with Institutional Human Research Ethics Approval (Behavioural & Social Sciences Ethical Review Committee, University of Queensland), ethical code project number 2012000869 and approved by the Behavioural & Social Sciences Ethical Review Committee, University of Queensland.

### 2.5. Data Analysis

Statistical analyses were carried out using the software package R [69]. Relationships between the socio-demographic indicators and each of the four measures of NR (NR Score, NR Self, NR Perspective, NR Experience; as four separate response variables) were tested using independent-samples *t* tests and analyses of variance, with effect sizes indicated by Cohen’s *d* and partial *η*-squared respectively. Highest formal qualification, work status, income, gender, language, presence of children in the home and age were treated as ordinal factors. Ordinal regression models (with a cumulative link function) were then used to test the association between the NR measures as predictors of depression, anxiety, stress and self-reported health. We first tested the association ignoring all possible covariates, and then included the other above-mentioned socio-demographic indicators and health-related variables as predictors. We checked for multicollinearity between predictors using the variance inflation factor (VIF). All variables had VIF < 2, which was sufficiently low to include them in the same model, except for the NR variables. Consequently, we included each as a predictor in its own separate model for each health variable because of the high level of correlation between the NR subscales (e.g., for depression we constructed four models, which included all covariates, and one each of NR Score, NR Self, NR Perspective, and NR Experience). We tested for zero-order correlations between the NR measures and the other predictors, between the NR measures and health variables, and between key significant predictor variables and health measures.

## 3. Results

Nature relatedness differed significantly across a number of socio-demographic characteristics (Table 1). People over 45 years had significantly higher scores across all NR measures in comparison to those less than 45 years. Women had higher scores than did men for all NR measures aside from NR Experience, where men had significantly higher scores. Those in the middle income quartiles had higher NR Self scores compared to those in the highest income quartile, but with very low effect sizes. People who did not have children living at home had higher NR scores for all scales. Those who completed a trade/diploma had higher NR Score, NR Self and NR Experience in comparison to those with those who had completed secondary school, again with very small effect sizes. Those speaking English at home had higher scores across all NR measures. People who did not work tended to have higher scores for the overall NR scale and NR Self compared with those who worked full time, with part-time workers reporting higher scores than full-time workers for NR Self (Table 1). See Supplementary Material Tables S2 and S3 for regression models assessing the relationship between socio-demographic variables and NR measures.

People with higher NR Score and NR Experience scores were more likely to report better self-reported health, and those with higher NR Experience were also more likely to report fewer symptoms of depression, anxiety and stress. Perhaps surprisingly, people with higher NR Self were more marginally likely to report poorer self-reported health, although the effect was rather weak (Table 2).

Accounting for the range of covariates, the association between NR Experience and better self-reported health remained significant, although NR Experience was no longer associated with fewer symptoms of depression, anxiety and stress. People with higher NR Score, NR Self and NR Perspective reported increased symptoms of stress, and those with higher NR Self also reported higher levels of depression and anxiety (Table 3). Significant associations were found between all nature relatedness measures and social cohesion, between three of the four nature relatedness measures and exercise, but there were no significant associations between any of the nature relatedness measures and BMI (Supplementary Materials Tables S2–S8).

## 4. Discussion

Using a community sample from Brisbane, Queensland we assessed differences in nature relatedness scores among different socio-demographic groups. We then investigated whether nature relatedness was associated with self-reported mental and physical ill health, including controlling for socio-demographic and other health-related characteristics that are potentially important covariates. People with higher scores on the Nature Relatedness scale were more likely to be older, female, not have children living at home, speak English at home and not work. In testing associations between NR measures and health variables, three of the four NR measures were associated with at least one measure of reduced ill health. Strikingly, people with higher NR Experience were significantly less likely to experience symptoms of depression, anxiety, stress and self-reported ill health. When potentially confounding variables were accounted for, NR Experience remained significantly associated with better self-reported health, but the associations with negative mental health were no longer significant. NR Score, NR Self and NR Perspective were associated with increased stress when socio-demographic and health-related variables were accounted for.

There are several possible explanations for these findings, and they suggest some fruitful directions for future research. Our finding that older people and females tend to report higher nature relatedness is consistent with some other studies of connectedness to nature [47,57,58]. The increase in nature relatedness for older people may reflect a cohort effect in which older people have had distinctly different experiences with nature throughout their lives compared with those born after the mid-1960s. It was during the 1960s that television and car ownership became more common in households, potentially leading to reduced time outdoors during leisure and recreation time [70]. Over the previous decades there has also been an escalation in the use of personal computers and engagement in social media associated with decreased time in natural settings [71]. It is also possible that a person’s orientation to nature changes across the life course; for example, the opportunities and motivations associated with spending time in natural settings for caregivers of young children are likely to be very different than those with adult children or in retirement [9,72]. These life changes may help explain why having children at home was associated with lower nature relatedness, with other caregiving tasks possibly taking a higher priority than engagement with and appreciation of nature. However, longitudinal research to consider causal pathways is needed to disentangle whether other explanations may be at play.

Our finding that females reported higher nature relatedness is consistent with most other studies, in which females tend to report greater environmental concern, behavior and attitudes than males, although notably this does not hold for specific subgroups, for example women in China appear to show lower environmental concern than do men [73,74]. In our study, while females had higher overall nature relatedness scores, males had higher scores on NR Experience, reflecting comfort and enjoyment of being in nature. Again, these differences are consistent with findings that women experience and use nature settings differently than men, particularly in relation to perceptions of safety [56].

It is unclear why people who do not speak English at home report lower nature relatedness. The construct of ‘nature relatedness’ is likely to differ across cultures and settings, given evidence of cross-cultural differences in landscape preferences, type of use and motivations to visit outdoor settings [75,76]. Although there is consistent evidence of high levels of concern about the environment and support for protection of the environment in cross-cultural studies over the previous two decades [77], there is substantial evidence of different types of environmental concern across countries and ethnic groups, with the ‘structure’ or focus of the concern markedly different across these diverse contexts [52]. To date, there has been little specific focus on the extent and nature of differences in nature relatedness between people from different cultural backgrounds [45]; most studies have recruited participants from western, high income countries, although recent research has also confirmed the positive association between nature relatedness and well-being in people from Japanese and Russian cultures [78].

We surmise there may be a particularly important role for NR Experience—the dimension of nature relatedness that concerns physical familiarity, comfort with and enjoyment in natural settings—given its association with reduced ill health across each of the health measures. NR Experience was also the only measure of nature relatedness that remained significantly associated with reduced physical ill health when potentially confounding demographic and health-related variables were included in the analysis. NR Experience is a more behaviorally focused measure of nature relatedness than the more psychological orientation of NR Self and NR Perspective, with items referring to experiential engagement in nature activities such as enjoying ‘being outdoors’ and ‘digging in the earth’. Given that evidence to date does not provide strong support for the premise that nature relatedness moderates the effect of nature contact on well-being [18,79], a key pathway to better health for people with higher NR Experience may be through their time spent in natural settings, which in turn provides people with the opportunity to obtain diverse health-related benefits. Indeed, higher scores on NR Experience may be associated with ‘exposure’ to nature across the life course [27].

The marked changes in the associations between nature relatedness measures and health variables when the broad range of socio-demographic and health-related variables were included in the analysis points to the need for additional understanding of causal pathways and complex interactions between a person’s orientation to nature and his/her life stage, health, psychological characteristics and social context. While our analysis accounts for health-related variables that could provide an intermediary pathway between nature relatedness and health (e.g., exercise, social cohesion), the potential complexity of underlying mechanistic pathways means we remain some way from a robust understanding of the impact of nature relatedness on health. For example, people may engage in high levels of exercise in built settings or experience high levels of social cohesion unrelated to activities connected to green space or natural settings.

Our finding that overall nature relatedness, as well as the subscales NR Self and NR Perspective, were all associated with increased stress (involving chronic non-specific arousal such as feeling agitated or having difficulty relaxing) was unexpected. Additional research is needed to investigate whether this finding can be replicated. One hypothesis is that people with greater environmental concern may experience increased psychological stress or distress related to the escalation of environmental degradation locally and globally. During the year prior to data collection there had been a major flood in Brisbane, as well as widespread dismantling of environmental regulation by the Queensland state government [80], both of which plausibly could contribute to additional stress related to environmental concern [81]. There is a small but growing literature regarding the emotional distress related to awareness of climate change, and consequences of environmental degradation more generally [81,82,83,84,85].

That higher scores on the NR Self subscale were associated with increased stress when the covariates were not included in the analysis, and also increased symptoms of depression and anxiety when the covariates were accounted for, further highlights the need for additional understanding of potentially negative aspects to dimensions of nature relatedness. Several questions in the NR Self subscale concern awareness of how one’s actions affect the environment, awareness of environmental issues, and concerns around the suffering of animals, all of which might be associated with increased negative emotions. Several studies have found negative or non-significant relationships between nature relatedness and the personality trait of neuroticism [41,43], although other research has found associations between neuroticism and environmental values [86] and environmental concern [87]. These inconsistent findings suggest that the specific psychological processes or dimensions inherent in different types of psychological orientation to nature should be carefully considered in future research investigating relationships between positive or negative emotions and nature relatedness, alongside careful consideration of the socio-demographic characteristics of sub-groups of people with these different psychological orientations.

### 4.1. Strengths and Limitations

This study provides a unique examination of the relationships between dimensions of nature relatedness and four health-related variables, using data from a large stratified sample of respondents. The study is of course somewhat limited by its cross-sectional design, which precludes drawing conclusions about the direction or pathways of causation; indeed it is possible that those who have better mental or physical health differ in their psychological capacity to connect not just with humans but also landscapes and living organisms.

Recent research has considered ways in which the construct of nature relatedness may also be linked with other unmeasured variables influencing the relationships with health measures; suggesting that linkages between nature relatedness and psychological well-being may be mediated by individual characteristics such as meaning in life [88] and spirituality [89]. Prospective longitudinal studies are required that carefully consider the causal pathways between nature-related orientations and behaviors (e.g., psychological connection to nature and exposure to nature—including frequency, duration, type of nature activity, quality of the nature setting) and a range of psychological and physical health outcomes across both the short and longer term. Including psychological orientation to nature in the systematic evaluation of health behavior interventions in natural settings (e.g., green exercise or ‘mindfulness in nature’ programs) will help shed light on the role of nature relatedness in moderating and mediating health outcomes.

Stratification of our sample enhanced representativeness of the population, although recruitment through a market research company may have introduced an unknown form of sampling bias. The sample was recruited from one Australian city, and findings may not be generalizable to other populations. Specifically, the participants were living within a Western high income context, a warm climate with substantial nearby green space conducive to outdoor activities, and relatively high environmental concern [90]. The use of self-report measures risks recall or response bias that could influence participant responses. The measure of depression, anxiety and stress was not a clinical diagnostic tool, rather it aimed to provide a measure of emotional experiences, including different characteristics of anxiety and depression, over the previous week [62]. The additional use of other measurement approaches such as implicit measures of nature relatedness [91], clinical diagnoses of anxiety or depression [25] or biomarkers of variation in stress levels [14] might enhance the validity of our findings. The use of single-item measures of health assisted to reduce response burden and enabled the data collection across a wide range of variables. Nonetheless, single item self-report measures such as exercise and BMI inevitably risk oversimplification.

A key consideration for interpreting the results laid out in this paper is the potential for over-adjustment bias. This issue arises where covariates are included in the models that are on the causal pathway from an influencing factor to an effect [92]. For example, this could be the case in our study if nature relatedness affected social cohesion, which in turn affected a health variable. While there is likely some small amount of bias present in this study, we expect that these effects are relatively minor. This is supported by the finding that we see similar directional results in the zero-order correlations to those outlined in the full models.

### 4.2. Future Research

The findings highlight the need for a more fine-grained understanding of the different dimensions of nature relatedness, and their associations with physical and mental health for diverse subpopulations. The mixed findings suggest that some dimensions of this psychological orientation—particularly a strong emotional connection to nature and awareness of environmental degradation—may be associated with negative emotions with the potential to contribute to ill health for some individuals. Further understanding is needed of the nature of any disbenefits, for whom, and in what circumstances, along with exploration of the potential for other societal benefits. For example, increasing environmental connection and concern may provide a critical catalyst for environmental behavior, given escalating ecosystem degradation globally and the urgent need to take action to promote socially and ecologically sustainable societies [93,94,95].

Careful attention to differences in nature relatedness across social groups, including people from different socioeconomic backgrounds, gender, life stage and cultural groups, is imperative in future research. Cross cultural consideration of nature relatedness is needed to understand if and how the construct of nature relatedness holds meaning for people from different ethnic and socio-cultural backgrounds, including the development of scales that are appropriate for cross-cultural research. In the Australian context, Indigenous Australian conceptualizations of nature and its inextricable linkages with health hold promise as a pathway to addressing health inequities as well as environmental issues [96].

Investigation of individuals’ connections to different forms of nature is also needed. Urban nature exists in many disparate forms—from private gardens to natural features on verges, parking lots and railway embankments—all of which have the potential to be valued and have salutogenic effects [97,98]. To date, research on nature relatedness has typically focused on ‘nature’ in general; understanding the ‘lived experience’ of connectedness to nature or ‘place attachment’ and considering how individuals relate to specific ecological features or places important in their lives will aid in developing more context-specific understanding of this construct, with implications for its potential to foster health and well-being [47,99,100].

The findings suggest that programs to foster the dimension of nature relatedness involving enjoyment of and comfort in natural settings may offer the most promising way forward as potential public health initiatives, although knowledge about how to foster nature relatedness is not yet well-developed. Childhood experiences in nature are frequently suggested as important in cultivating nature relatedness, especially engagement with ‘expansive’ or ‘wild’ natural settings [41,101]. However, little is known about the extent to which nature relatedness changes over the longer term in the context of formative experiences for children, or indeed across different life stages. Longitudinal studies showing the predictive value of nature relatedness to child health and development, as well as experimental studies of approaches to increase nature relatedness via families, childcare, schools and other contexts, are needed. There is promising research suggesting that school-based education programs involving direct engagement with nature may result in increased connection to nature, as well as findings that child-led immersion in nature settings may also promote increased parental engagement in nature activities with their children [102]. There is also potential to promote nature relatedness through immersive environmental education programs and wilderness expeditions [18,103,104] and psychological approaches involving self-reflective practices such as curiosity about emotions, attitudes, values and thoughts while being exposed to natural elements [105]. Consideration of intervention elements beyond engagement in the nature setting itself is also worthy of consideration, such as key role models (parents, grandparents, teachers, community leaders) communicating and inspiring connection to nature [106] or creative arts that involve engaging with natural elements [107]. Of critical importance is understanding how to foster nature relatedness among the population as a whole—particularly subgroups experiencing social disadvantage—to avoid perpetuating and amplifying health inequities.

Finally, there may be new ways forward in considering ‘nature relatedness’ in deeper or expanded ways that move beyond relatively constrained conceptions of humans in a relationship with ‘nature’ confined to landscapes and living organisms. This could encompass a broader relational understanding of self as part of a planetary ecosystem that involves not just living systems but built, material environments and social relationships. There is burgeoning theoretical work on the need for cultural change in which our sense of self is embedded within wider global systems in order to respond to critical challenges, such as the growing gaps between advantaged and disadvantaged groups within and across nations, and ecological imperatives such as biodiversity loss, pollution and climate change [108].

## 5. Conclusions

This paper has systematically considered the relationships between a broad range of socio-demographic factors and dimensions of nature relatedness, and presented some possible explanations of the development of higher nature relatedness in specific social groups. Additionally, the study investigated relationships between affective, cognitive and experiential dimensions of nature relatedness and mental and physical ill health. The nature relatedness orientation in which people feel pleasure in spending time in natural settings showed particular promise as a potential protective factor against high levels of depression, anxiety and poor self-reported health. The associations between negative mental health and the dimension of nature relatedness involving an internalized sense of identification with nature highlights the need to further understand the potentially negative aspects of nature relatedness for mental health. A critical next step is systematic longitudinal and experimental research that disentangles causal pathways between different dimensions of nature relatedness and the health outcomes experienced, including careful consideration of diversity between subpopulations and the contexts in which they live.

## Figures and Tables

**Table 1:** Comparing Nature Relatedness across Socio-Demographic Groups.

Variable	Number in Group	NR Score ^a^		NR Self ^a^		NR Perspective ^a^		NR Experience ^a^	
		Average	Std Dev	Average	Std Dev	Average	Std Dev	Average	Std Dev
Age									
<45	952	3.38	0.59	3.29	0.72	3.60	0.70	3.24	0.83
≥45	586	3.64	0.59	3.67	0.71	3.77	0.72	3.45	0.74
Cohen’s d			0.441		0.538		0.232		0.269
Gender									
Male	809	3.43	0.60	3.35	0.71	3.53	0.73	3.43	0.77
Female	729	3.52	0.61	3.51	0.75	3.81	0.66	3.19	0.82
Cohen’s d			0.150		0.220		0.414		−0.290
Income quartile									
First	342	3.46	0.59	3.44	0.75	3.68	0.72	3.24	0.79
Second	363	3.50	0.60	3.49	0.75	3.69	0.67	3.29	0.82
Third	351	3.54	0.59	3.48	0.72	3.71	0.71	3.40	0.80
Fourth	482	3.42	0.62	3.34	0.72 *,^a^	3.60	0.73	3.33	0.80
Effect size (ANOVA)			N.S.		0.007		N.S.		N.S.
Presence of children <16 years in home									
Not present	1129	3.51	0.62	3.47	0.76	3.69	0.71	3.36	0.82
Present	409	3.38	0.54	3.34	0.66	3.57	0.69	3.20	0.76
Cohen’s d			0.223		0.174		0.177		0.191
Educational attainment									
Secondary school not completed	145	3.44	0.57	3.43	0.67	3.65	0.71	3.22	0.82
Secondary school completed	333	3.39	0.58	3.33	0.72	3.63	0.67	3.21	0.82
Trade/Diploma or equivalent	405	3.54	0.61 *,^b^	3.50	0.77 *^b^	3.68	0.70	3.42	0.73 **,^b^
University degree	472	3.49	0.64	3.43	0.76	3.69	0.75	3.34	0.86
Post-graduate degree	183	3.48	0.56	3.49	0.67	3.62	0.71	3.29	0.78
Effect size (ANOVA)			0.002		0.002		N.S.		0.002
Primary language spoken at home									
English	1325	3.50	0.61	3.44	0.74	3.68	0.71	3.37	0.80
Other language	213	3.32	0.54	3.37	0.72	3.55	0.67	2.98	0.77
Cohen’s d			6.756		5.700		6.308		5.123
Work status in survey week									
No work	392	3.54	0.58	3.53	0.71	3.72	0.67	3.32	0.78
Part-time	738	3.47	0.60	3.44	0.73	3.64	0.70	3.30	0.80
Full-time	408	3.43	0.63 **^c^	3.32	0.76 ***^c^, **^d^	3.63	0.76	3.33	0.83
Effect size (ANOVA)			0.004		0.011		N.S.		N.S.

*Note.* NR scale/subscales range from 1–5. Effect size for ANOVA analyses are indicated by partial eta-squared. ^a^ Compared with 2nd & 3rd income quartiles. ^b^ Compared with secondary school completed. ^c^ Compared with no work. ^d^ Compared with part-time work. * *p* < 0.05; ** *p* < 0.01; *** *p* < 0.001.

**Table 2:** Relationships between Nature Relatedness and Four Health Variables (From Ordinal Regression Models, Cumulative Link), Without Accounting for Covariates (Zero Order Correlations).

Health Variable	Coefficient (Standard Error)			
	NR Score	NR Self	NR Perspective	NR Experience
Depression	−0.11(0.07)	−0.02(0.06)	−0.04(0.06)	−0.17(0.06) **
Anxiety	−0.08(0.08)	0.05(0.06)	−0.04(0.07)	−0.17(0.06) **
Stress	−0.03(0.07)	0.00(0.00)	0.07(0.06)	−0.12(0.06) *
Self-reported health	0.26(0.08) ***	−0.15(0.06) *	−0.03(0.07)	0.38(0.06) ***

*Note.* Co-efficients are unstandardized. * *p* <.05; ** *p* < 0.01; *** *p* < 0.001.

**Table 3:** Relationships between Nature Relatedness and Four Health Variables (From Ordinal Regression Models, Cumulative Link), Accounting for Covariates.

Health Variable	Coefficient (Standard Error)			
	NR Score	NR Self	NR Perspective	NR Experience
Depression	0.07(0.08)	0.15(0.06) *	0.05(0.06)	−0.07(0.06)
Anxiety	0.13(0.08)	0.24(0.06) ***	0.05(0.06)	−0.04(0.06)
Stress	0.17(0.08) *	0.19(0.06) **	0.15(0.06) *	−0.02(0.06)
Self-reported health	0.13(0.08)	0.08(0.07)	−0.08(0.07)	0.24(0.07) ***

*Note.* Covariates were included in all models and full results are shown in the Supplementary Material. For depression, anxiety and stress, the coefficients were derived from ordinal regression models with a cumulative link function. Coefficients are unstandardized. * *p* < 0.05; ** *p* < 0.01; *** *p* < 0.001.

## Supplementary Materials

The following are available online at http://www.mdpi.com/1660-4601/15/7/1371/s1. Table S1: Socio-demographic variables, Table S2: Results from ordinal regression models (Cumulative link) assessing the relationship between socio-demographic predictor variables and nature relatedness score and nature relatedness self (Zero Order Correlations), Table S3: Results from ordinal regression models (Cumulative link) assessing the relationship between socio-demographic predictor variables and nature relatedness perspective and nature relatedness experience (Zero Order Correlations), Table S4: Results from ordinal regression models (Cumulative link) assessing the relationship between socio-demographic predictor variables and symptoms of depression, Table S5: Results from ordinal regression models (Cumulative link) assessing the relationship between socio-demographic variables and symptoms of anxiety, Table S6: Results from ordinal regression models (Cumulative link) assessing the relationship between socio-demographic predictor variables and symptoms of stress, Table S7: Results from ordinal regression models (Cumulative link) assessing the relationship between socio-demographic predictor variables and respondent’s assessment of their own health, Table S8: Results from ordinal regression models (Cumulative link) assessing the relationship between predictor variables and respondent’s assessment of their own health. These models consider each predictor separately without other variables (i.e., zero order correlations). The three predictors shown are important predictors of the health variables measured here.
