# Supplementary material for: Is Nature Relatedness Associated with Better Mental and Physical Health?
Source: Int J Environ Res Public Health. 2018 Jun 29;15(7):1371. doi: 10.3390/ijerph15071371 (PMC6069224; doi:10.3390/ijerph15071371)
Supplement: Supplementary file 1 [file ijerph-15-01371-s001.pdf]

International Journal of
Environmental Research and Public Health

Online Supplementary Materials

Table S1. Socio-Demographic Variables

Variable	Description
Age	Age range (12 response options from 18-20 years, then five year increments until 70+ years). For analysis this was split into two categories (<45, ≥45), based on evidence that health changes significantly at this point (the risk of being diagnosed with hypertension begins to increase steeply at age 45 years (Australian Bureau of Statistics, 2013), and the prevalence of affective mood disorders such as depression begins to decline in Australia at about 45 (Slade, Johnston, Teesson, Whiteford, Burgess, Pirkis, & Saw, 2009).
Sex	Male or female; for analysis purposes male = 1, female = 2.
Income quartile	Personal income selected from categories defined based on the income question provided in the Australian census (categories included weekly income of: nil or negative; $1-$199; $200-$299; $300-$399; $400-$599; $600-$799; $800-$999; $1000-$1249; $1250-$1499; $1500-$1999; $2000+). For analysis purposes each respondent was grouped into the income quartile from the actual population that they fell within.
Presence of children <16 years in home	The presence or absence of people living in a respondent's home who were under 16 years at the time of the survey.
Educational attainment	The highest formal educational qualification achieved by the respondent, grouped into five categories (1 = *school not completed*; 2 = *school completed*; 3 = *trade/diploma or equivalent*; 4 = *university degree*; 5 = *post-graduate degree*).
Primary language spoken at home	An indication of the language primarily spoken at home. For analysis purposes 0 = *English*, 1 = *not English*.
Work status in survey week	Respondents provided information on the number of hours worked in a normal week, and this was grouped into no-work (0 hours), part-time (<40 hours), or full-time (>40hours).

Table S2. Results From Ordinal Regression Models (Cumulative Link) Assessing the Relationship Between Socio-Demographic Predictor Variables and Nature Relatedness Score and Nature Relatedness Self (Zero Order Correlations)

	NR Score			NR Self		
	Estimate	Std err	Z value	Estimate	Std err	Z value
Age	0.767***	0.092	8.285	0.923***	0.093	9.881
Gender (1 male, 2 female)	0.240**	0.089	2.706	0.393***	0.089	4.408
Income Linear	-0.018	0.097	-0.208	-0.124	0.087	-1.427
Income quartile Quadratic	-0.211*	0.089	-2.366	-0.232**	0.090	-2.588
Income quartile Cubic	-0.097	0.091	-1.063	-0.020	0.092	-0.217
Children <16 in home	-0.400***	0.099	-4.053	-0.282**	0.098	-2.866
Work hours Linear	-0.222*	0.087	-2.551	-0.343***	0.087	-3.936
Work hours Quadratic	0.024	0.072	0.330	-0.040	0.072	-0.558
Educational attainment Linear	0.134	0.125	1.072	0.141	0.124	1.135
Educational attainment Quadratic	-0.064	0.116	-0.554	0.088	0.115	0.769
Educational attainment Cubic	-0.133	0.099	-1.345	-0.113	0.098	-1.155
Educational attainment Quartic	0.182*	0.089	2.047	0.234**	0.090	2.606
Language		0.125	-4.477		0.126	-1.239
(0, English, 1 not English)	-0.560***			-0.156		
Frequency of exercise ≥30mins	0.102***	0.021	4.828	0.076***	0.021	3.635
BMI	0.227	0.210	1.081	0.227	0.210	1.081
Social cohesion	0.590***	0.086	6.861	0.605***	0.086	7.068

* $p < .05$; ** $p < .01$; *** $p < .001$.

Table S3. Results From Ordinal Regression Models (Cumulative Link) Assessing the Relationship Between Socio-Demographic Predictor Variables and Nature Relatedness perspective and Nature Relatedness Experience (Zero Order Correlations)

	NR perspective			NR Experience		
	Estimate	Std err	Z value	Estimate	Std err	Z value
Age	0.421***	0.092	4.57	0.430***	0.091	4.714
Gender (1 male, 2 female)	0.684***	0.090	7.608	-0.512***	0.089	-5.729
*Income (*Linear	-0.112	0.088	-1.277	0.203*	0.087	2.323
Income quartile Quadratic	-0.144	0.089	-1.614	-0.127	0.089	-1.419
Income quartile Cubic	-0.107	0.091	-1.178	-0.113	0.092	-1.233
Children <16 in home	-0.343***	0.100	-3.424	-0.368***	0.099	-3.7
Work hours Linear	-0.125	0.087	-1.435	0.037	0.087	0.426
Work hours Quadratic	0.069	0.072	0.950	0.064	0.072	0.880
Educational attainment Linear	0.012	0.127	0.093	0.194	0.128	1.517
Educational attainment Quadratic	-0.060	0.117	-0.512	-0.216	0.117	-1.848
Educational attainment Cubic	-0.139	0.100	-1.404	-0.151	0.100	-1.516
Educational attainment Quartic	-0.017	0.089	-0.194	0.177*	0.088	2.001
Language		0.127	-2.819		0.127	-6.644
(0, English, 1 not English)	-0.357**			-0.847***		
Frequency of exercise ≥30mins	0.023	0.021	1.082	0.149***	0.027	7.062
BMI	-0.026	0.212	-0.125	0.308	0.214	1.441
Social cohesion	0.301***	0.086	3.513	0.536***	0.086	6.224

* $p < .05$; ** $p < .01$; *** $p < .001$.

Table S4. Results From Ordinal Regression Models (Cumulative Link) Assessing the Relationship Between Socio-Demographic Predictor Variables and Symptoms of Depression

	Model 1 [NR Score]	Model 2 [NR Self]	Model 3 [NR Perspective]	Model 4 [NR Experience]
Age	-0.02(<0.01)***	-0.02(<0.01)***	-0.01(<0.01)***	-0.01(<0.01)***
Gender (1 male, 2 female)	-0.15(0.09)	-0.16(0.09)	-0.16(0.09)	-0.17(0.09)
Income quartile Linear	-0.25(0.1)*	-0.24(0.1)*	-0.25(0.1)*	-0.26(0.1)*
Income quartile Quadratic	-0.18(0.09)	-0.17(0.09)	-0.18(0.09)	-0.19(0.09)*
Income quartile Cubic	-0.04(0.09)	-0.04(0.09)	-0.04(0.09)	-0.05(0.09)
Children <16 in home	-0.11(0.05)*	-0.1(0.05)*	-0.11(0.05)*	-0.11(0.05)*
Work hours Linear	-0.28(0.1)**	-0.28(0.1)**	-0.28(0.1)**	-0.28(0.1)**
Work hours Quadratic	0.11(0.07)	0.11(0.07)	0.11(0.07)	0.11(0.07)
Educational attainment Linear	0.25(0.14)	0.23(0.14)	0.26(0.14)	0.29(0.14)*
Educational attainment Quadratic	-0.07(0.12)	-0.06(0.12)	-0.07(0.12)	-0.08(0.12)
Educational attainment Cubic	0.16(0.1)	0.17(0.1)	0.16(0.1)	0.16(0.1)
Educational attainment Quartic	-0.02(0.09)	-0.02(0.09)	-0.02(0.09)	-0.02(0.09)
Language (0, English, 1 not English)	-0.16(0.14)	-0.17(0.14)	-0.16(0.14)	-0.2(0.14)
Frequency of exercise ≥30mins	-0.12(0.02)***	-0.12(0.02)***	-0.12(0.02)***	-0.11(0.02)***
BMI	1.13(0.24)***	1.13(0.24)***	1.13(0.24)***	1.14(0.24)***
Social cohesion	-0.66(0.09)***	-0.68(0.09)***	-0.65(0.09)***	-0.63(0.09)***
NR Score	0.07(0.08)			
NR Self		0.15(0.06)*		
NR Perspective			0.05(0.06)	
NR Experience				-0.07(0.06)

Note. Each model included a different NR measure [shown in square brackets]. The sum of the scores on the Depression subscale was used as the response variable. Coefficients are provided with the standard error in brackets, **and are unstandardized**. Work hours, income and educational attainment were ordered factor variables, so the polynomial contrasts were used to assess the pattern of the relationship.
* $p < .05$; ** $p < .01$; *** $p < .001$.

Table S5. Results From Ordinal Regression Models (Cumulative Link) Assessing the Relationship Between Socio-Demographic Predictor Variables and Symptoms of Anxiety

	Model 1 [NR Score]	Model 2 [NR Self]	Model 3 [NR Perspective]	Model 4 [NR Experience]
Age	-0.02(<0.01)***	-0.02(<0.01)***	-0.02(<0.01)***	-0.02(<0.01)***
Gender (1 male, 2 female)	-0.07(0.09)	-0.08(0.09)	-0.07(0.1)	-0.07(0.09)
Income quartile Linear	-0.19(0.11)	-0.18(0.11)	-0.2(0.11)	-0.2(0.11)
Income quartile Quadratic	-0.26(0.09)**	-0.25(0.09)**	-0.27(0.09)**	-0.27(0.09)**
Income quartile Cubic	-0.07(0.09)	-0.06(0.09)	-0.07(0.09)	-0.07(0.09)
Children <16 in home	-0.09(0.05)	-0.08(0.05)	-0.09(0.05)	-0.09(0.05)
Work hours Linear	-0.22(0.11)*	-0.22(0.11)*	-0.21(0.11)*	-0.21(0.11)*
Work hours Quadratic	0.1(0.07)	0.11(0.07)	0.1(0.07)	0.11(0.07)
Educational attainment Linear	0.11(0.14)	0.08(0.14)	0.13(0.14)	0.15(0.14)
Educational attainment Quadratic	0.1(0.12)	0.1(0.12)	0.09(0.12)	0.09(0.12)
Educational attainment Cubic	0.06(0.1)	0.06(0.1)	0.06(0.1)	0.05(0.1)
Educational attainment Quartic	0(0.09)	0(0.09)	0.01(0.09)	0.01(0.09)
Language (0, English, 1 not English)	0.32(0.14)*	0.3(0.14)*	0.31(0.14)*	0.29(0.14)*
Frequency of exercise >30mins	-0.09(0.02)***	-0.09(0.02)***	-0.08(0.02)***	-0.08(0.02)***
BMI	1.01(0.24)***	1.03(0.24)***	1.01(0.24)***	1.01(0.24)***
Social cohesion	-0.5(0.09)***	-0.53(0.09)***	-0.48(0.09)***	-0.46(0.09)***
NR Score	0.13(0.08)			
NR Self		0.24(0.06)***		
NR Perspective			0.05(0.06)	
NR Experience				-0.04(0.06)

Note. Each model included a different NR measure [shown in square brackets]. The sum of the scores on the Anxiety subscale was used as the response variable. Coefficients are provided with the standard error in brackets, and are unstandardized. Work hours, income and educational attainment were ordered factor variables, so the polynomial contrasts were used to assess the pattern of the relationship.
* $p < .05$; ** $p < .01$; *** $p < .001$.

Table S6. Results From Ordinal Regression Models (Cumulative Link) Assessing the Relationship Between Socio-Demographic Predictor Variables and Symptoms of Stress

	Model 1 [NR Score]	Model 2 [NR Self]	Model 3 [NR Perspective]	Model 4 [NR Experience]
Age	-0.02(<0.01)***	-0.02(<0.01)***	-0.02(<0.01)***	-0.02(<0.01)***
Gender (1 male, 2 female)	0.02(0.09)	0.01(0.09)	0(0.09)	0.03(0.09)
Income quartile Linear	-0.17(0.1)	-0.16(0.1)	-0.18(0.1)	-0.18(0.1)
Income quartile Quadratic	-0.13(0.09)	-0.12(0.09)	-0.12(0.09)	-0.14(0.09)
Income quartile Cubic	0.12(0.09)	0.12(0.09)	0.12(0.09)	0.11(0.09)
Children <16 in home	0.05(0.05)	0.05(0.05)	0.05(0.05)	0.04(0.05)
Work hours Linear	0(0.1)	0(0.1)	0(0.1)	0(0.1)
Work hours Quadratic	0.11(0.07)	0.12(0.07)	0.11(0.07)	0.12(0.07)
Educational attainment Linear	0.25(0.14)	0.24(0.14)	0.26(0.14)	0.29(0.14)*
Educational attainment Quadratic	-0.01(0.12)	-0.01(0.12)	-0.01(0.12)	-0.03(0.12)
Educational attainment Cubic	0.13(0.1)	0.12(0.1)	0.12(0.1)	0.12(0.1)
Educational attainment Quartic	0.04(0.09)	0.04(0.09)	0.05(0.09)	0.04(0.09)
Language (0, English, 1 not English)	-0.1(0.14)	-0.12(0.14)	-0.1(0.14)	-0.12(0.14)
Frequency of exercise >30mins	-0.04(0.02)*	-0.04(0.02)*	-0.04(0.02)	-0.03(0.02)
BMI	0.75(0.24)**	0.76(0.24)**	0.76(0.24)**	0.76(0.24)**
Social cohesion	-0.61(0.08)***	-0.62(0.08)***	-0.6(0.08)***	-0.57(0.08)***
NR Score	0.17(0.08)*			
NR Self		0.19(0.06)**		
NR Perspective			0.15(0.06)*	
NR Experience				-0.02(0.06)

Note. Each model included a different NR measure [shown in square brackets]. The sum of the scores on the Stress subscale was used as the response variable. Coefficients are provided with the standard error in brackets, **and are unstandardized** Work hours, income and educational attainment were ordered factor variables, so the polynomial contrasts were used to assess the pattern of the relationship.
* $p < .05$; ** $p < .01$; *** $p < .001$.

Table S7. Results From Ordinal Regression Models (Cumulative Link) Assessing the Relationship Between Socio-Demographic Predictor Variables and Respondent's Assessment of Their Own Health

	Coefficient (standard error)			
	Model 1 [NR Score]	Model 2 [NR Self]	Model 3 [NR Perspective]	Model 4 [NR Experience]
Age	0(<0.01)	0(<0.01)	0.00(<0.01)	0(<0.01)
Gender (1 male, 2 female)	-0.24(0.1)*	-0.21(0.1)*	-0.15(0.1)	-0.17(0.1)
Income quartile Linear	0.3(0.11)**	0.29(0.11)**	0.31(0.11)**	0.29(0.11)**
Income quartile Quadratic	0.13(0.1)	0.12(0.1)	0.17(0.1)	0.13(0.1)
Income quartile Cubic	-0.07(0.1)	-0.08(0.1)	-0.08(0.1)	-0.07(0.1)
Children <16 in home	0.01(0.05)	0(0.05)	0.01(0.06)	0.01(0.05)
Work hours Linear	0.34(0.11)**	0.34(0.11)**	0.33(0.11)**	0.36(0.11)**
Work hours Quadratic	0.2(0.08)*	0.2(0.08)*	0.23(0.08)**	-0.21(0.08)**
Educational attainment Linear	0.13(0.15)	0.16(0.15)	0.07(0.15)	0.1(0.15)
Educational attainment Quadratic	0.01(0.13)	0(0.13)	-0.00(0.13)	0.02(0.13)
Educational attainment Cubic	0(0.11)	0(0.11)	0(0.11)	0(0.11)
Educational attainment Quartic	-0.05(0.1)	-0.05(0.1)	-0.06(0.1)	-0.06(0.1)
Language (0, English, 1 not English)	-0.18(0.14)	-0.21(0.14)	-0.25(0.14)	-0.12(0.14)
Frequency of exercise >30mins	0.26(0.02)***	0.27(0.02)***	0.26(0.02)***	0.25(0.02)***
BMI	-2.62(0.26)***	-2.64(0.26)***	-2.74(0.26)***	-2.63(0.26)***
Social cohesion	0.49(0.09)***	0.53(0.09)***	0.37(0.08)***	0.47(0.09)***
NR Score	0.13(0.08)			
NR Self		0.08(0.07)		
NR Perspective			-0.08(0.07)	
NR Experience				0.24(0.07)***

Note. Each model included a different NR measure [shown in square brackets]. Work hours, income and educational attainment were ordered factor variables, so the polynomial contrasts were used to assess the pattern of the relationship.
* $p < .05$; ** $p < .01$; *** $p < .001$.

Table S8. Results From Ordinal Regression Models (Cumulative Link) Assessing the Relationship Between Predictor Variables and Respondent's Assessment of Their Own Health. These Models Consider Each Predictor Separately Without Other Variables (i.e. Zero Order Correlations). The Three Predictors Shown Are Important Predictors of the Health Outcomes Measured Here.

	Coefficient (standard error)			
	Model 1 [Depression]	Model 2 [Anxiety]	Model 3 [Stress]	Model 4 [Self-reported health]
Frequency of exercise >30mins	-0.12(0.02)***	-0.08(0.02)***	-0.04(0.02)	-0.28(0.02)***
BMI	0.92(0.22)***	0.58(0.22)**	0.31(0.22)	2.74(0.24)***
Social cohesion	-0.70(0.09)***	-0.56(0.09)***	-0.63(0.09)***	-0.55(0.09)***
